# A genome-wide cis-regulatory element discovery method based on promoter sequences and gene co-expression networks

**DOI:** 10.1186/1471-2164-14-S1-S4

**Published:** 2013-01-21

**Authors:** Zhen Gao, Ruizhe Zhao, Jianhua Ruan

**Affiliations:** 1 Department of Computer Science, The University of Texas at San Antonio, One UTSA Circle, San Antonio, TX 78249, USA; 2 Ronald Reagan High School, 19000 Ronald Reagan, San Antonio, TX 78258, USA

## Abstract

**Background:**

Deciphering *cis*-regulatory networks has become an attractive yet challenging task. This paper presents a simple method for *cis*-regulatory network discovery which aims to avoid some of the common problems of previous approaches.

**Results:**

Using promoter sequences and gene expression profiles as input, rather than clustering the genes by the expression data, our method utilizes co-expression neighborhood information for each individual gene, thereby overcoming the disadvantages of current clustering based models which may miss specific information for individual genes. In addition, rather than using a motif database as an input, it implements a simple motif count table for each enumerated *k*-mer for each gene promoter sequence. Thus, it can be used for species where previous knowledge of *cis*-regulatory motifs is unknown and has the potential to discover new transcription factor binding sites. Applications on *Saccharomyces cerevisiae *and *Arabidopsis *have shown that our method has a good prediction accuracy and outperforms a phylogenetic footprinting approach. Furthermore, the top ranked gene-motif regulatory clusters are evidently functionally co-regulated, and the regulatory relationships between the motifs and the enriched biological functions can often be confirmed by literature.

**Conclusions:**

Since this method is simple and gene-specific, it can be readily utilized for insufficiently studied species or flexibly used as an additional step or data source for previous transcription regulatory networks discovery models.

## Background

The advance of experimental technology, including complete genome sequencing, high-throughput expression profiling [1-3] and binding-site mapping [4-6], has made the computational approach of studying the *cis*-regulatory networks (CRN) more attractive. A widely used model is to cluster genes based on their expression profile and then using motif finding algorithms [7-14] or motif enumerators to find the over-represented sequences within each cluster [15-18]. However, the correlation between gene clusters and motifs is imprecise because of the complex nature of regulation, as not all genes within a cluster share a common motif and the same motif can be found in gene promoters in other clusters. Importantly, these clustering based models are not suitable to model the expression of each individual gene. To overcome this limitation, [19] and [20] have proposed a linear regression model to predict statistically significant motifs. While a fascinating approach, it assumes that the number of occurrences of motifs in a promoter is linearly correlated with the gene expression, where motifs are identified by simply enumerating all *k*-mers. From an entirely different angle, [21] provided an approach that screened genomic sequences against a database of putative regulatory motifs, evaluating the contribution of the occurrences of the motif on the gene expression by comparing the expression profiles of genes containing this motif against those that do not. This method therefore requires knowledge about the putative motifs of that species, which may not always be available. Another major direction of motif finding and CRN discovery focuses on comparative genomics, also referred to as phylogenetic footprinting (PF) [22-34]. It assumes that the *cis*-regulation is conserved over evolution; thus, it does not need gene co-expression data as input to determine the sets of co-regulated genes. PF is a powerful method and has gained impressive success for the prediction of conserved regulatory elements. However, the main drawback of this method is that it cannot find species-specific regulatory elements.

Here, we propose a simple approach for inferring CRNs that avoids the common limitations mentioned above. Using genomic promoter sequences and gene expression data as input, our method utilizes the gene expression data in a novel way. In contrast to the approach favored by previous clustering based methods, which clusters genes by co-expression information and finds over-represented motifs for each cluster, our method first builds a gene co-expression network, and then searches for putative cis-regulatory elements that are enriched in the neighborhood for each individual gene on the network. In other words, our method utilizes the gene expression profiles in an individual gene motivated fashion. In addition, by using a simple enumerated *k*-mer counter to find the motif information within the promoter sequences, this approach needs little knowledge of the species-based putative motifs and requires few assumptions about the model by which elements of motif counts affect gene expression. It is a simple and versatile model for motif discovery and CRN finding. Thus, we hope it can be easily used for species with little previous *cis*-regulatory knowledge or be flexibly used for previous CRNs discovery approaches as an additional step. An *in silico *evaluation on *Saccharomyces cerevisiae *and *Arabidopsis *has been performed. Compared to a phylogenetic footprinting (PF) based method on several datasets, our method shows comparable or even better prediction accuracy. Furthermore, the top ranked *cis*-regulatory clusters uncovered by our approach for the two species are evidently functionally co-regulated, and the regulatory relationships between the motifs and the enriched biological functions can often be confirmed by literature.

## Methods

### Cis-regulatory network construction

The input of our method includes a list of promoter sequences and a gene-gene co-expression network (see Data sources). The *m *promoter sequences are viewed as 'background' and the *n *genes of the co-expression network are the target genes whose CRNs we want to study, where the n target genes are a subset of the *m *background genes. The gene-gene co-expression network is represented by an adjacency matrix, **A **= 〈*a_ij_*〉_*n *× *n*_, where *a_ij _*= 1 if there is an edge between gene *i *and gene *j*, and 0 otherwise. For convenience, we let *a_ii _*= 1 for all *i*. Here the number of target genes *n *is from co-expression networks. The reason why we choose *n *target genes out of *m *is that in order to ensure a high-quality co-expression data, only the genes with the highest variations in the stress response data set will be chose to build co-expression network.

We first count the number of occurrences for each *k*-mer, where *k *= 6 in this study, on each of the *m *promoter sequences. Let **C **= 〈*c_ij_*〉_*m *× *l *_be the *k*-mer occurrence table, where *l *= 4*^k ^*is the total number of candidate motifs (*k*-mers). Let **D **= 〈*d_ij_*〉_*n *× *l *_be a matrix derived from **C **such that each row of **D **corresponds to a row in **C **for the same gene's promoter, and the order of the genes in **D **is equivalent to that in **A**.

Next, for each gene *g *present in the co-expression network, we identify its neighbors in the network, *π_g _*= {*i*|*a_gi _*= 1}, and retrieve the corresponding rows from **D**, defined as **T***^g ^*= 〈*t_ij_*〉 = 〈*d_sj_*〉, where *s *∈ *π_g_*.

Finally, we compute the significance for the *j*-th *k*-mer being over-represented in the neighborhood of gene g using either the cumulative hypergeometric test, or student's t-test. With the cumulative hypergeometric test, the *p*-value is calculated as

(1) $$p_{gj}=\overset{\text{min}\left(K,q\right)}{\underset{r=x}{\sum }}\frac{\left(\begin{array}{c} K\\ r \end{array}\right)\left(\begin{array}{c} m-K\\ q-r \end{array}\right)}{\left(\begin{array}{c} m\\ q \end{array}\right)}$$

where *x *= |{*i*|**T***^g ^*= 〈*t_ij_*〉, *t_ij _*> 0}| is the number of genes within gene *g*'s neighborhood (including gene *g *itself) that have at least one occurrence of motif *j, K *= |{*i*|*c_ij _*> 0}| is the number of such genes in the background (whole genome), and *q *= |*π_g_*| is the number of neighbors for gene *g *in the network. Intuitively, the cumulative hypergeometric *p*-value is the probability of drawing at least *x *of a possible *K *items in *q *drawings without replacement from a group of *m *objects.

On the other hand, with the student's t-test, the *p*-value is calculated by performing a non-paired two-sample t-test to compare the average occurrence of motif *j *in the neighborhood of *g *against that of the genes not in the neighborhood of *g*.

From the *p*-value matrix **P **= 〈*p_ij_*〉_*n *× *l*_, a score matrix **S **= 〈*s_ij_*〉_*n *× *l *_can be computed by *s_ij _*= log_10 _*p_ij_*. The values in **S **range from 0 to +∞, where a greater number indicates a more significant regulatory relationship of a gene-motif pair. To cope with numerical precision limitations, all scores larger than 40 are converted to 40.

At last, the *cis*-regulatory network, represented by a bipartite graph **R **= (*U, V, E, W*) can be derived from **S**, where *U *is the set of genes, *V *is the set of motifs, *E *is the set of regulatory relationships, and *W *is the associated edge weights, defined by *w_ij _*= *s_ij _*for any edge. To ensure statistical significance and biological relevance, a cutoff is applied so that (*U_i_, V_j_*) ∈ *E *if and only if *s_ij _*>*cutoff*. In our study, for evaluation purposes, *cutoff *is set to 2, corresponding to a *p*-value threshold at 0.01. For visualization and biological analysis, *cutoff *is set to a much larger value (17 for yeast and 30 for *Arabidopsis*, see Results).

### Evaluation of the predicted cis-regulatory network

To directly evaluate the predicted *cis*-regulatory network is difficult. We evaluated the performance of our algorithm indirectly in two ways.

#### Co-regulatory network based evaluation

First, following the idea in [34], we evaluate the CRNs based on co-regulatory networks. To this end, we first determine the similarity (Pearson correlation coefficient) between each pair of genes based on their motif scores. Subsequently, a co-regulatory network, **N**, is constructed by connecting genes whose similarity scores are above a certain threshold. This co-regulatory network is then compared with some reference networks to determine the performance of our method. The key idea here is that if two genes share many regulatory elements, they are likely to be functionally related, and would share more edges with a reference network (see Data sources), which also captures functional relevance of genes. As the reference network and our predicted network may not have the same size, we first limit both networks to contain the same set of genes. We then compared the two networks using a set of standard metrics, defined as follows.

• True positive (*TP*) is the number of edges in both **N**_1 _and **N**_2_: *TP *= | *Q*_1 _∩ *Q*_2_|;

• False positive (*FP*) is the number of edges in **N**_1 _but not **N**_2_: $FP=\;|Q_{1}\cap \bar{Q_{2}}|$;

• True negative (*TN*) is the number of non-edges in both **N**_1 _and **N**_2_: $TN=\;|\bar{Q_{1}}\cap \bar{Q_{2}}|$;

• False negative (*FN*) is the number of edges in **N**_2 _but not **N**_1_: $FN=\;|Q_{2}\cap \bar{Q_{1}}|$;

• Precision, also referred to as the positive predictive value (PPV), is the ratio between *TP *and number of edges of **N**_1_: $PPV=\frac{TP}{\left|Q_{1}\right|}=\frac{TP}{TP+FP}$;

• Recall, also referred to as sensitivity or true positive rate (TPR), is the ratio between *TP *and number of edges of **N**_2_: $TPR=\frac{TP}{\left|Q_{2}\right|}=\frac{TP}{TP+FN}$.

Here **N**_1 _and **N**_2 _represent the predicted co-expression network and the reference co-expression network, respectively, *Q_i _*represent the set of edges in **N***_i_*, and $\bar{Q_{i}}$ represents the set of edges in the inverse graph of **N***_i_*.

#### Model based evaluation

Second, we evaluate the CRN using a completely different strategy. Our idea is that, if the CRN is correct and complete, we should be able to use it to model gene transcriptional level changes with a high accuracy. Therefore, given a set of gene expression microarray data, we attempt to construct a linear regression model for each microarray to predict the expression levels for each gene using the linear combination of the gene's motif scores. Importantly, in order to perform an unbiased evaluation, the gene expression microarray data used for evaluating the predicted *cis*-regulatory network should be different from the gene expression data that we have used for constructing the gene co-expression network or *cis*-regulatory network (see Data sources).

Formally, let *e_g _*be the logarithm base two of the ratio of mRNA levels between two conditions for gene *g*, we model *e_g _*by $e_{g}=\sum_{i=1}^{k}\;\beta_{i}w_{ig}+c$. This formulation is identical to the popular model proposed by [19], except that they used motif occurrences (i.e., the **D **matrix), while we use the motif significance score (i.e., the **S **score and with some statistical cutoff). As the number of *k*-mer motifs (4096) we have is larger than the number of genes (3000), we apply a simple feature selection by only including the top *q *(*q *« 3000) motifs that have the highest correlation between motif significance scores and gene expression levels, and perform linear regression only using these top motifs. To measure the accuracy of the model, we calculated the root mean squared error (RMSE) of the linear model, as well as the Pearson correlation coefficient (PCC) between the predicted expression levels and the actual values. A higher PCC or a lower RMSE indicates a better prediction accuracy and therefore a biologically more relevant CRN.

#### Competing methods

We compare our method with two alternative methods. The first method is based on a naive model where we simply score each promoter sequence by counting the number of occurrences of each *k*-mer (*k *= 6 as in our main model). It is expected that such a simple method will su er from high false positive (i.e., a given *k*-mer may not be functional) and false negative (e.g., a motif instance may be missed due to mismatch). Nevertheless, this is the model used in most methods attempting to model gene transcriptional changes (e.g. [19,20]). The second method was proposed by [34], where they used phylogenetic footprinting to identify putative cis-regulatory elements in yeast *Saccharomyces cerevisiae *by discovering over-represented motifs in the promoters of their orthologs in 19 *Saccharomycetes* species. In their method, cis-regulatory elements are represented by dyads, i.e. pair of trinucleotides separated by a spacing comprised between 0 and 20 bp. The output of their method is similar to the gene-motif *p*-value matrix and we applied the same logarithm transformation as in our method so that a higher score means a more significant regulatory relationship.

### Data sources

#### Promoter sequences

Promoter sequences for *Saccharomyces cerevisiae *(budding yeast) are downloaded from RSA tools [35]. The promoter sequence for each target gene is defined as 500 base pairs upstream from its transcription start site (TSS), or the whole intergenic region between the TSS and the coding sequence of the upstream gene, whichever is shorter.

Promoter sequences for *Arabidopsis thaliana *are downloaded from TAIR (arabidopsis.org). Promoter sequences are defined as 1000 base pairs upstream to the first annotated nucleotide of the gene (regardless of UTR or coding), according to TARI10 assembly.

#### Microarray data and gene co-expression networks

To construct a gene co-expression network, we used gene expression microarray data from [36], which contains the yeast gene expression data in response to a variety of environmental changes. This data set contains 173 arrays, and as in most previous studies, we selected the top 3000 genes with the highest variances [36]. After quantile normalizing the expression data, we constructed a co-expression network using the method described in [37]. Brie y, we first computed the Pearson correlation coefficient between the expression profiles of every pair of genes, and then ranked the correlation coefficients for each gene separately. Two genes are connected by an edge if the correlation between their expression profiles is ranked above a certain threshold within both genes' rankings. The threshold is determined automatically from an analysis of the resulting network's topological properties as described in [37]. The optimal rank threshold chosen is 120. Previous studies showed that such rank-based co-expression network can produce biologically more meaningful functional modules, especially for gene modules that are weakly co-expressed or conditionally co-expressed [37].

To evaluate the predicted *cis*-regulatory network for yeast, we used a separate yeast gene expression microarray data from [38], which contains 77 arrays measuring gene expression under various cell cycle phases.

Gene expression microarray data for *Arabidopsis *is obtained from AtGenExpress, which includes more than 1391 microarrays for various growth conditions, developmental stages, and tissues of *Arabidopsis *[39,40]. The gene co-expression network is constructed similarly as above. The optimal rank threshold chosen is 100.

#### Reference networks

The choice of a proper reference network is crucial. In this work, we choose to evaluate our predicted CRN for *Saccharomyces cerevisiae *using two reference networks that are constructed also based on the principle of co-regulation. The first reference network, the annotated regulon network, is constructed by linking pairs of genes belonging to the same annotated regulon that have been proved by low-throughput experiments [41,42]. The second reference network, known as the co-binding network, is derived from high-throughput ChIP-chip data [6], where we link any pair of genes that are shown to be bound by at least one common TF in the ChIP-chip data (with *p*-value < 0.001). The annotated regulon network is smaller and less complete than the co-binding network, but probably more accurate. Table 1 shows some basic statistics of the networks. It is worth noting that the same networks were used by the competing method [34] in their paper to evaluate their co-regulatory networks.

**Table 1:** Size of the comparison networks

	Annotated co-regulation network		Chip-chip co-binding network		**similarity matrix of ref **[34]	Cis-similarity matrix
	
	Nodes	Edges	Nodes	Edges	Nodes	Nodes
Original size	612	10,599	2,397	178,202	3,146	3,000
Similarity matrix of ref [34]	446	5,816	1,491	77,597	3,146	1,909
Cis-similarity matrix	467	6,554	1,439	73,982	1,909	3,000
Intersect with both	360	3,984	1,006	39,693	1,909	1,909

This table shows the size of the intersections of the networks that are restricted by the nodes (genes names) of the sub-networks. 'Cis-similarity matrix' represents our predicted co-regulatory similarity matrix.

## Results and discussion

### The predicted cis-regulatory network (CRN) of *Saccharomyces cerevisiae*

#### The predicted co-regulatory network has a good accuracy

**Figure 1:**
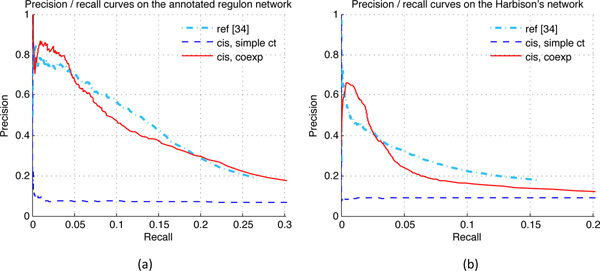
**Co-regulatory network based evaluation results**. Evaluation result of three models were shown - the reference model developed by Brohée, *et al*., 2011 (denoted by 'ref [34]'), the naive model with simple *k*-mer counts (denoted by 'cis, simple ct'), and our main model with both sequence information and gene expression neighborhood information from the gene co-expression network (denoted by 'cis, coexp'). **(a) **The comparison on the annotated regulon network. **(b) **The comparison on the Harbison's ChIP-chip based co-binding network.

First, as shown in Figure 1, the model that simply includes the promoter sequences information (denoted by 'cis, simple ct') has a very poor performance. The precision (*PPV*) is almost always lower than 20%. The red solid line shows the improvement by including both the promoter sequences and the neighborhood information from the co-expression networks, indicating that including the gene neighborhood information is critical for this method.

Compared to [34] using the annotated regulon network, our precision is higher when the recall is less than 0.04 or greater than 0.2 (Figure 1a). The comparison using the ChIP-chip co-binding network [6] shows that, when the recall is less than 0.025, our precision is better (Figure 1b). The advantage for the top predictions indicates that our method is better for species-specific predictions. These results show that the prediction accuracy of our approach is comparable with the phylogenetic footprinting approach for co-regulatory network prediction.

Since the predicted co-regulatory networks under comparison are intersected with annotated-regulon network or Chip-chip co-binding network, the evaluation covers only a small part of the original predicted co-regulatory networks, so the real prediction accuracy cannot be fully represented by these evaluated results. Compared to the annotated-regulon network, the *PPV *of high-scoring prediction, with the score cutoff greater than 0.2, is higher than 50%. Thus, among the 90189 edges of the 3000 by 3000 original predicted co-regulatory network with cutoff = 0.2, there should be a large portion of edges that correspond to real co-regulations.

#### The predicted cis-regulatory network has a good correlation with the gene expression profiles

**Figure 2:**
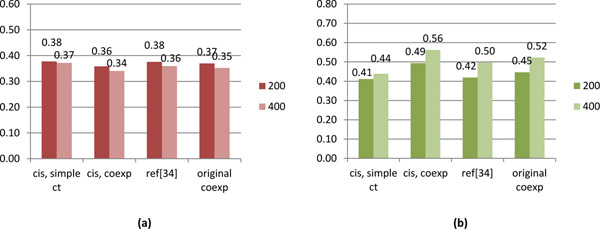
**Linear regression evaluation result**. It shows the RMSE (**a**) and the correlation (**b**) between the prediction values of the linear models (which are based on the *cis*-regulatory matrices) and the gene expression profiles. The three *cis*-regulatory element finding approaches are the naive model with simple *k*-mer counts (denoted by 'cis, simple ct'), our main model with both sequence information and gene expression neighborhood information (denoted by 'cis, coexp'), and the reference model developed by Brohée, *et al*., 2011 (denoted by 'ref [34]'). The fourth column shows the modeling result for the gene expressions by the original input co-expression network of our method. The legend on the right of each bar chart indicates the number of top *k*-mers used to build the linear model and predict the expression level (see Methods). A lower RMSE or a higher correlation indicates a better prediction accuracy.

We evaluated the predicted *cis*-regulatory network by using it to model the gene expression level changes, and then finding the accuracy of the predicted expression level. It is important to note that the reference microarray gene expression dataset [38] used for modeling here is different from the gene expression dataset [36] used for constructing the co-expression networks and *cis*-regulatory network. From Figure 2, it can be seen that our main model has the best result based on both the correlation and the root mean square error (RMSE). The result from the PF method ('Ref [34]') is significantly lower than our main method and only slightly better than the naive model. This is in sharp contrast to the evaluation results based on co-regulatory networks, where both methods significantly outperformed the naive method. One possible explanation is that while the PF method can predict well-conserved cis-regulatory elements with high accuracy, it will miss species-specific motifs, which are important to accurately model the gene expression levels. In addition, we attempted to predict the cell cycle gene expression data using the co-expression network (which was constructed from the stress-response data and a starting point of our algorithm) directly with the linear regression model. This result is shown by the fourth sets of bar in Figure 2. It shows that although the co-expression network performs the second best, it is still a little weak compared to our method.

Since our goal is to reveal the regulatory network, in other word, to find the relationship between the transcription factors and the target genes, the overall good quality of modeling gene expression data doesn't tell the best part of our approach. If we can find examples that the input gene expression profile cannot modeling the evaluating gene expression profile well, but our gene-motif score matrix can, that would show the value of our method. Thus, we provided the figure in additional file 1. The x axis shows the 77 gene expression conditions of the evaluating gene expression profile. The y axis shows the correlation between the predicted expression level and the true expression level. The red curve shows the results of our approach and the blue curve shows the results of the original input co-expression network of our approach. Both of the prediction results are using the top 400 features. From the figure, we can see that for some of the conditions, our model performs better than the original input co-expression profile. In addition, the modeling factors of our approach are the k-mers which uncover the potential TF binding sites.

#### The predicted cis-regulatory network for yeast is biologically relevant

**Figure 3:**
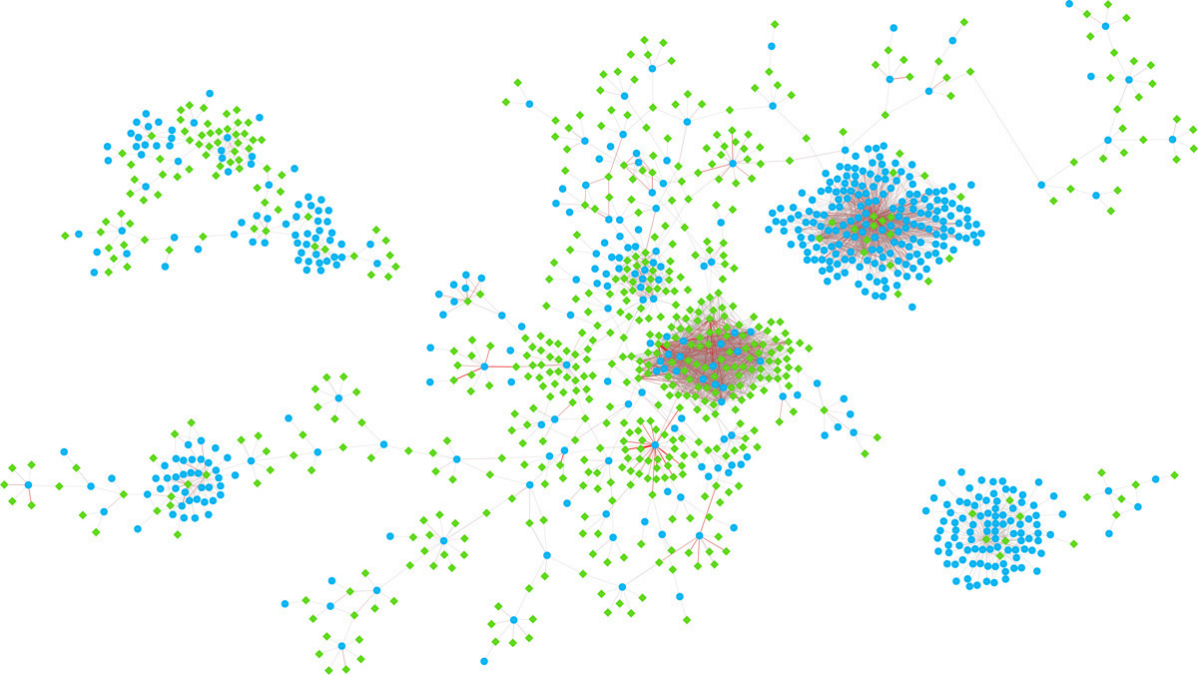
**A predicted *Saccharomyces cerevisiae* cis-regulatory network**. It is a whole *cis*-regulatory network of *Saccharomyces cerevisiae *predicted by this paper. Starting from the gene vs. motif significance score matrix, **S**, we use a score cutoff of 17 to produce the *cis*-regulatory network, **R**. The green diamonds represent 6-mer motifs and the blue ellipses represent genes. The color and width of the edges show the significance score. The color ranges from light gray to red (while the width ranges from 1 to 7), representing the significance score range from 17 to 126, respectively. The major clusters shown in this figure may have important biological meaning (see Figure 2 and Table 2).

**Figure 4:**
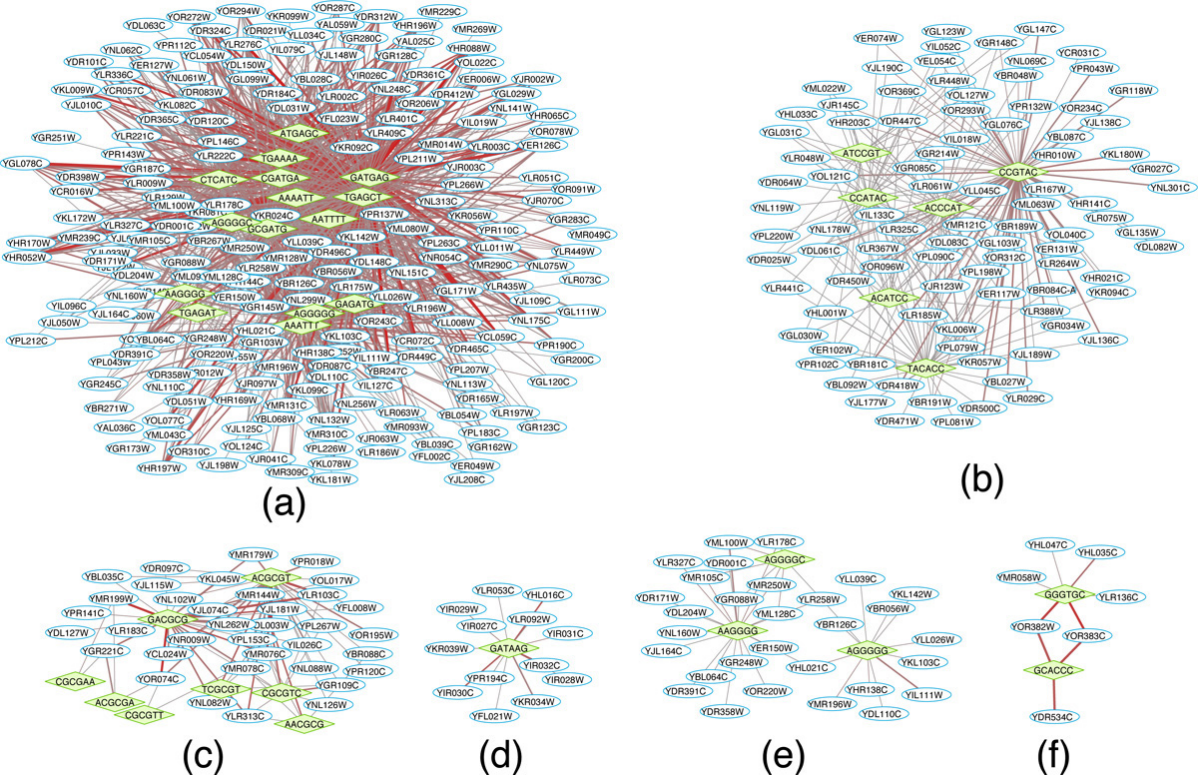
**Six representative predicted clusters of *Saccharomyces cerevisiae***. The green diamonds represent 6-mer motifs and the blue ellipses represent genes. The graph visual style is changed from Fig. 3 in order to show the gene names clear. The functional clusters discovered by the *DAVID Functional Annotation Clustering *tool within the six cluster are shown by Table 2.

We have performed a preliminary analysis to evaluate the biological significance of the predicted *cis*-regulatory networks. For efficiency, we only focused on the most significant portion of the network. Using a cutoff 17 on the the motif significance score matrix, **S **(see Methods), we produced a sparse CRN that contains several relatively large and dense clusters. Figure 3 and 4 shows the overall network topology and the six selected clusters, respectively.

Then, we analyzed the functions of genes in the discovered cis-regulatory clusters using the *DAVID Functional Annotation Clustering *tool. Table 2 shows the discovered *cis*-regulatory elements and annotated functional clusters for the six *cis*-regulatory modules shown in Figure 4. The significance of the functional annotations is measured by the Fisher's exact test for the enrichment level of the function within the cluster relative to the genome-wide genes with this function [43]. As shown, all six clusters are significantly enriched with some functional terms. More importantly, literature search confirmed that the *cis*-regulatory elements identified in each module matched the binding sites of transcription factors that are known to regulate the corresponding biological process. For example, the consensus motif regulating the subnetwork in Figure 4a, GATGAGC, resembles the well-known PAC motif (GATGAG), which has been shown recently to be the binding sites of two transcription factors Pbf1 and Pbf2 and regulates ribosome biogenesis [44]. The module in Figure 4b contains multiple *cis*-regulatory elements that appear to be sub-sequences of the Rap1-binding motif, CACCCRWACA [45], which is known to be present in most yeast ribosomal protein genes [46]. The *cis*-regulatory elements in Figure 4c include the known binding sites for Mbp1 (ACGCGT) and Swi4 (CGCGAA), two key regulators of yeast cell cycle [46]. The GATAAG motif in Figure 4d matches the binding sites of several GATA-family TFs, including Gat1, Gln3, Dal80, and Gzf3, all of which are known to be involved in nitrogen metabolism [46]. Figure 4e contains the well-known stress-response element (STRE, AAGGGG) bound by Msn2 and Msn4 proteins [45]. Finally, the GCACCC motif shown in Figure 4f matches perfectly with the binding sites of Aft1, which is known to be involved in iron utilization and homeostasis [46]. Therefore, the discovered *cis*-regulatory modules are functionally relevant.

**Table 2:** Discovered cis-regulatory clusters for *Saccharomyces cerevisiae*

d-clust	d-count	related 6-mers	Functional Cluster Name	f-count	P-val	
Fig. 4a	182	AAAATT, AAATTT, AATTTT, ATGAGC, CGATGA, CTCATC, GAGATG, GATGAG, GCGATG, TGAAAA, TGAGAT, TGAGCT, AAGGGG, AGGGGC, AGGGGG	nucleolus	120	2.90E-129	
			ribosome biogenesis	90	2.30E-108	
			ncRNA processing	111	1.70E-94	
			90S preribosome	45	4.50E-47	
			nuclear lumen	127	1.20E-97	

Fig. 4b	85	TACACC, CCGTAC, CCATAC, ATCCGT, ACCCAT, ACATCC	cytosolic ribosome	82	9.70E-131	
			ribosome	79	2.90E-128	
			structural constituent of ribosome	82	6.40E-111	
			cytosolic small ribosomal subunit	36	1.30E-51	

Fig. 4c	36	AACGCG, ACGCGA, ACGCGT, CGCGAA, CGCGTC, CGCGTT, GACGCG, TCGCGT	chromosome	16	6.00E-11	
			cell cycle	23	9.00E-13	
			mitotic sister chromatid cohesion	9	9.40E-12	
			DNA metabolic process	19	6.60E-11	

Fig. 4d	13	GATAAG	amide catabolic process	6	6.20E-13	
			purine metabolism	5	3.40E-11	

Fig. 4e	30	AAGGGG, AGGGGC, AGGGGG,	response to temperature stimulus	21	6.30E-25	
			response to abiotic stimulus	21	8.10E-21	
			vacuolar protein catabolic process	14	4.20E-16	

Fig. 4f	7	GCACCC, GGGTGC	iron transport	5	3.10E-09	
			iron ion transport	5	2.60E-08	
			siderophore transport	4	8.70E-08	

'd-clust' is the identifier for the discovered clusters; 'd-count' is the number of genes in the discovered cluster; 'f-count' is the number of genes in the functional clusters, which was discovered by previous well-organized experiments; 'P-value' is measured by Fisher's exact test p-value, which measures the enrichment level of the functions within the discovered cis-regulatory cluster relative to the genome.

### The predicted cis-regulatory network of Arabidopsis

**Figure 5:**
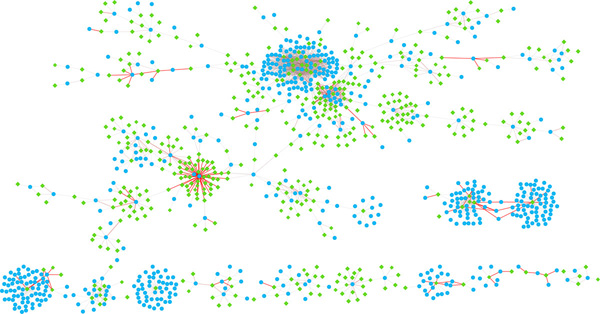
**A predicted Arabidopsis cis-regulatory network**. It is the graphical representation of the predicted *cis*-regulatory network, **R**, for *Arabidopsis*, with cutoff 30 on the score matrix, **S**. The color and the width of the edges indicate the significance score.

**Figure 6:**
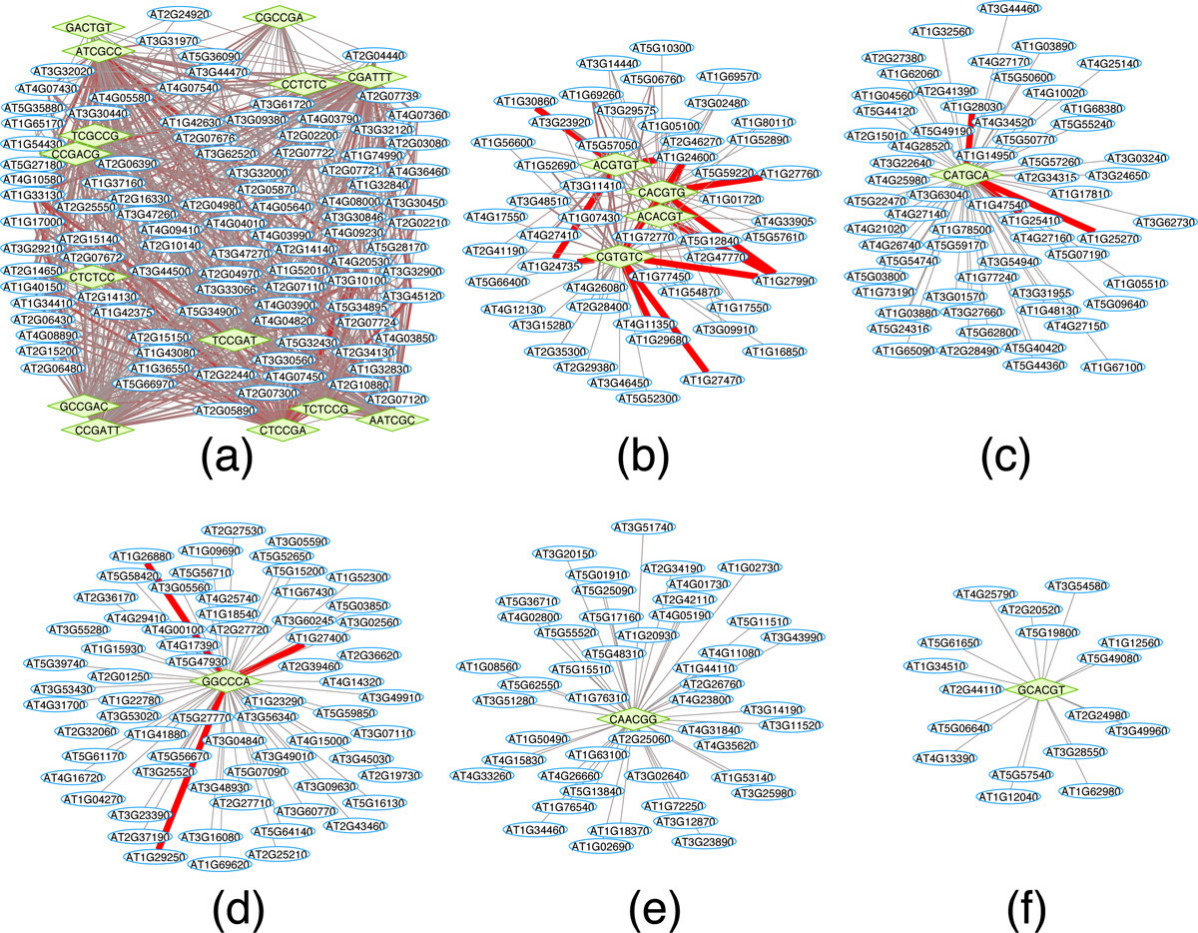
**Six representative predicted clusters of *Arabidopsis***. Refer to Table 3 for the discovered functional clusters within the six predicted cis-regulatory clusters.

Similar to the process of evaluating the predicted *cis*-regulatory network for *Saccharomyces cerevisiae*, using an *S *cutoff of 30, we determine the *cis*-regulatory network for Arabidopsis (Figure 5). For the six most interesting clusters (Figure 6), we present the discovered annotated functional clusters in Table 3 from the *DAVID Functional Annotation Clustering *tool. The Arabidopsis is much more poorly annotated compared to yeast, and much less is known about the *cis*-regulatory networks in Arabidopsis. Nevertheless, the *cis*-regulatory modules identified by our method still shows significant functional coherence. For example, several motifs in Figure 6b match to the core subsequence (CACGTG) of the well-known abscisic acid responsive element (ABRE) [47]. The CATGCA motif in Figure 6c is part of the RY-repeat element that is specifically required during seed development [47]. The motif GGCCCA in Figure 6d matches the UP1ATMSD motif that is over-represented in a list of up-regulated genes after main stem decapitation in Arabidopsis, many of which are predicted to function in protein synthesis [48]. Finally, genes in Figure 6e are involved in cell cycle and are regulated by the CAACGG motif, which contains the myb core motif found in the promoter of Arabidopsis cell cycle regulating cyclin B1:1 gene [47].

**Table 3:** Discovered cis-regulatory clusters for Arabidopsis

d-clust	d-count	related 6-mers	Functional Cluster Name	f-count	P-val	
Fig. 6a	95	AATCGC, ATCGCC, CCGACG, CCGATT, CCTCTC, CGATTT, CGCCGA, CTCCGA, CTCTCC, GACTGT, GCCGAC, TCCGAT, TCGCCG, TCTCCG	cysteine-type peptidase activity	8	4.60E-11	

Fig. 6b	50	ACACGT, ACGTGT, CACGTG, CGTGTC	response to water deprivation	11	1.50E-11	
			response to abscisic acid stimulus	12	7.90E-11	
			PP2C SIG	8	9.80E-11	
			Protein phosphatase 2C	8	1.20E-10	

Fig. 6c	61	CATGCA	nutrient reservoir activity	12	1.80E-18	
			Seed storage protein	7	6.50E-16	

Fig. 6d	66	GGCCCA	cytosolic ribosome	64	1.90E-103	
			cytosolic part	61	3.10E-99	
			ribosomal protein	65	3.00E-121	
			structural constituent of ribosome	65	8.10E-104	

Fig. 6e	47	CAACGG	cell cycle	11	7.60E-13	
			cell division	10	2.00E-12	

Fig. 6f	17	GCACGT	plant-type cell wall organization	8	9.80E-14	
			Extensin-like region	6	7.00E-13	
			structural constituent of cell wall	6	4.40E-11	

'd-clust' is the identifier for the discovered clusters; 'd-count' is the number of genes in the discovered cluster; 'f-count' is the number of genes in the functional clusters, which was discovered by previous well-organized experiments; 'P-value' is measured by Fisher's exact test p-value, which measures the enrichment level of the functions within the discovered cis-regulatory cluster relative to the genome.

## Conclusions

The method provided by this paper combined gene expression profiles and promoter sequences in a novel way. By including the neighborhood information from the gene expression profiles rather than clustering the genes, it does not neglect the information of each individual gene in the expression profiles. The accuracy of the predicted co-regulatory network was high when compared to the annotated regulon network, the ChIP-chip co-binding network, and outperformed a phylogenetic footprinting based method [34]. Additionally, by using the motif enumerator, it is more flexible for discovering cis-regulatory elements in various species, or for improving current CRN discovering methods. Compared to current methods using phylogenetic footprinting, this method is better for discovering species specific co-regulations.

The future work of this paper includes two directions. First, more advanced motif models could be used for improving the accuracy of regulatory network discovery. Currently, we just use the simplest approach in each step to show that the co-expression network-based approach can improve the specificity of cis-regulatory networks. For the 6-mer model, it may have more false positives compared with longer k-mer models. Later, we would like to try some state of the art approaches to identify cis-regulatory element. Second, better methods can be designed to identify genes that are not necessarily direct neighbors of a target gene, but are likely involved in similar biological processes. Finally, more tests could be done by applying this method on other species.

## Abbreviations

CRN: cis-regulatory network; PF: phylogenetic footprinting; PCC: Pearson correlation coefficient: RMSE: root mean squared error; TSS: transcription start site.

## Competing interests

The authors declare that they have no competing interests.

## Authors' contributions

ZG designed the study, performed the data analysis and drafted the manuscript. RZ assisted in the data analysis and writing. JR conceived of the study, participated in its design and coordination and helped to draft the manuscript. All authors read and approved the final manuscript.

## Declarations

The publication costs for this article were funded by NIH grant SC3GM086305.

This article has been published as part of *BMC Genomics *Volume 14 Supplement 1, 2013: Selected articles from the Eleventh Asia Pacific Bioinformatics Conference (APBC 2013): Genomics. The full contents of the supplement are available online at http://www.biomedcentral.com/bmcgenomics/supplements/14/S1.

## Supplementary Material

Additional file 1 **The comparison of prediction accuracy for gene expression between methods**. Click here for file
