# Supplementary material for: A genome-wide cis-regulatory element discovery method based on promoter sequences and gene co-expression networks
Source: BMC Genomics. 2013 Jan 21;14(Suppl 1):S4. doi: 10.1186/1471-2164-14-S1-S4 (PMC3549801; doi:10.1186/1471-2164-14-S1-S4)
Supplement: Additional file 1 — The comparison of prediction accuracy for gene expression between methods. [file 1471-2164-14-S1-S4-S1.pdf]

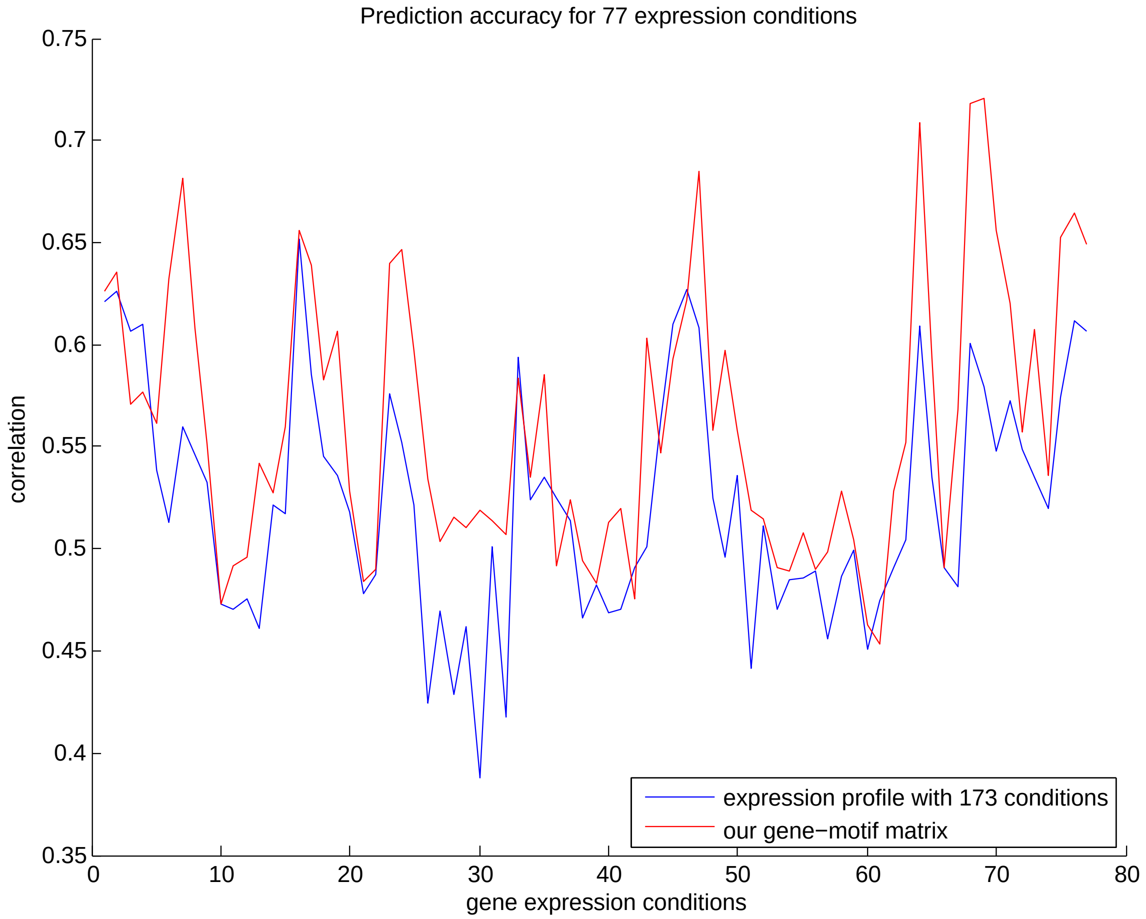

The x axis shows the 77 gene expression conditions of the evaluating gene expression profile. The y axis shows the correlation between the predicted expression level and the true expression level. The red curve shows the results of our approach and the blue curve shows the results of the original input co-expression network of our approach. Both of the prediction results are using the top 400 features.
